# Mitigation of image distortion during mechanical testing within a dynamic stereo x-ray system

**DOI:** 10.3389/fbioe.2025.1571639

**Published:** 2025-05-19

**Authors:** David N. Paglia, John M. Chomack, David V. Herlihy, Charlene Wetterstrand, Yazan Kadkoy, Roman Duchnycz, Patrick Kelly, J. Patrick O’Connor, Susan E. D’Andrea, Jason T. Maikos

**Affiliations:** ^1^ Department of Orthopaedics, Rutgers-New Jersey Medical School, Newark, NJ, United States; ^2^ Prosthetics and Sensory Aids Service, Veterans Affairs New York Harbor Healthcare System, New York, NY, United States; ^3^ Narrows Institute for Biomedical Research and Education, Inc., Brooklyn, NY, United States; ^4^ Department of Kinesiology, University of Rhode Island, Kingston, RI, United States

**Keywords:** biplanar fluoroscopy, dynamic stereo x-ray, skin strain, mechanical testing, magnetic field, magnetic field shielding

## Abstract

**Background:**

Dynamic stereo x-ray (DSX) permits *in-vivo* skin strain quantification with high accuracy. Validation of image-derived strain can be performed via mechanical testing inside a DSX capture volume while simultaneously comparing strain measurements. However, electromagnetic mechanical testing systems (eMTSs) emit magnetic fields that affect DSX image formation components and cause image distortion. This study presents a custom solution to redirect this magnetic field from the DSX capture volume to mitigate image distortion.

**Methods:**

A MuMETAL-lined box contoured to the test frame was developed to divert the magnetic field from the DSX test space. To assess the design, a radiopaque object was placed in the eMTS with shielding and within the DSX capture volume at either 65 or 103 cm from the image intensifiers (IIs) while the speed of the eMTS actuator was systematically increased from 0.1 to 10 mm/s during image collection. Root mean square error (RSME) was calculated over 1,000 frames for each test condition.

**Results:**

Results indicated a proportional change in RSME with increasing distance and decreasing speed. At 65 cm, higher actuator speeds (10 mm/s) produced the largest RSME (0.11 mm), significantly higher than the control test. At 103 cm, RSME was below 0.05 mm for all speeds.

**Conclusion:**

While closer distance to the IIs and higher actuator speeds produced larger RSME, results indicated that RSME for all experimental conditions fell below the established RSME associated with DSX marker tracking. The MuMETAL-lined box therefore mitigated DSX image distortion caused by the eMTS regardless of distance to the IIs and actuator speed.

**Clinical Trial Registration:**

clinicaltrials.gov, identifier NCT05287646.

## 1 Introduction

Determining accurate mechanical parameters of skin has proven to be a challenge in the biomechanics field (Graham et al., 2019). Skin is a highly nonlinear, viscoelastic, anisotropic, multi-layered tissue with distinct physical and mechanical properties that vary by body location (Graham et al., 2019; Ni Annaidh et al., 2012; Ottenio et al., 2015; Jor et al., 2013; Geerligs et al., 2011; Remache et al., 2018; Sandford et al., 2013). Traditional techniques to measure the mechanical behavior of the skin have been extensively performed under various conditions using tensile (Ni Annaidh et al., 2012; Ottenio et al., 2015), indentation (Geerligs et al., 2011; Jor et al., 2013), suction, and torsion testing (Salter et al., 1993). Additionally, mechanical testing can also be performed under *in-vivo*, *in-situ*, or *in-vitro* conditions. These standard tissue testing techniques have provided critical data to better characterize the tissue mechanics of skin (Geerligs et al., 2011; Jor et al., 2013; Ni Annaidh et al., 2012; Remache et al., 2018; Ottenio et al., 2015), though many drawbacks persist that potentially alter the mechanical behavior of the skin. For example, *in-vitro* testing offers simpler experimental setups under controlled conditions, but the loss of physiological conditions and pre-tension tends to reduce the integrity of the sample (Griffin et al., 2016). *In vivo* testing preserves important anatomical conditions but is typically limited to small deformations to reduce the invasive nature of testing (Tran et al., 2007). Additionally, skin tends to display different mechanical behavior during dynamic conditions (Sandford et al., 2013) which is often not addressed using standard testing techniques. Therefore, innovative approaches are needed to characterize the mechanical behavior of skin, particularly during dynamic activities.

**FIGURE 1:**
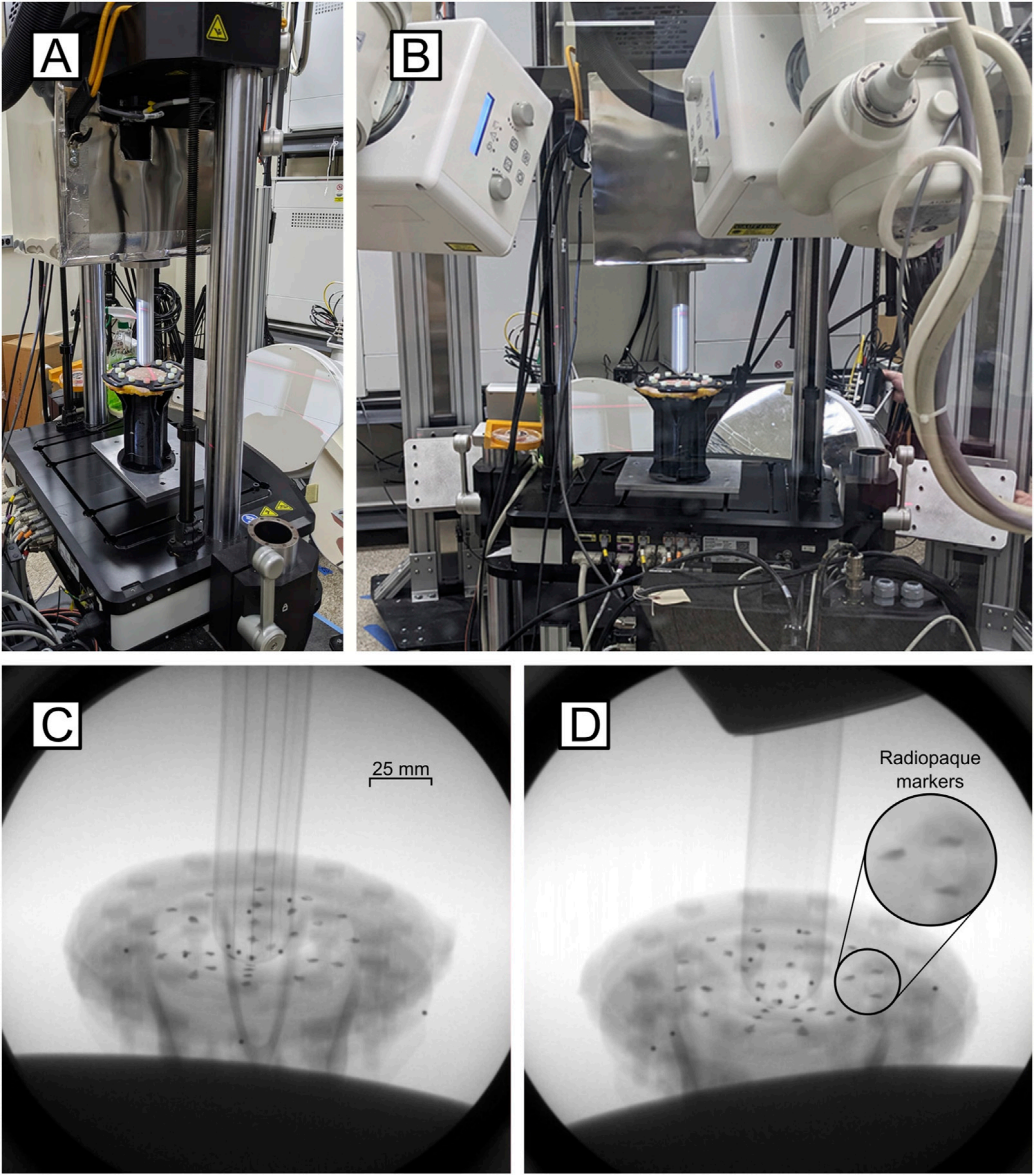
Validation apparatus setup. **(A)** The mechanical testing system interfaces with the DSX System. **(B)** Full view of the testing setup showing testing inclination. **(C)** Inline and **(D)** offset x-ray images of the skin painted with radiopaque markers during extrusion testing.

With the recent evolution of dynamic stereo x-ray (DSX) (Bey et al., 2008; Bey et al., 2006; Brainerd et al., 2010; Zhang et al., 2013; Dall'Ara et al., 2022; Maikos et al., 2021), this advanced imaging technology offers the potential to evaluate *in-vivo* tissue strains with high levels of accuracy and repeatability (Bey et al., 2008; Bey et al., 2006; Brainerd et al., 2010; Zhang et al., 2013; Maikos et al., 2021), specifically during dynamic activities. Previous unvalidated studies have used DSX to measure *in-vivo* tissue strain in prosthetic sockets for individuals with lower limb loss (Gale et al., 2020; Papaioannou et al., 2010). However, measuring tissue strain using DSX image-derived strain calculations requires validation against gold standard measurements prior to widespread implementation. Validation can be performed via traditional tensile or extrusion skin testing using a mechanical testing system inside a DSX capture volume (Figure 1), which offers a secondary method of imaged movement calculation for tissue strain. The load cell from the mechanical testing system can directly detect force values at the point of application and the strain along a tissue testing surface can be calculated based on the geometry of the skin sample. DSX imaging can be used for simultaneous marker tracking to determine the change in distance between radiopaque markers applied to the skin. However, electromagnetic mechanical testing systems (eMTSs) emit a local magnetic field. DSX image formation components (i.e., IIs) are known to be sensitive to magnetic fields. While the x-ray IIs are designed with electromagnetic shielding built into the housing, magnetic fields exceeding those shielding limits may cause damage to the IIs. The eMTS located within the DSX field creates a magnetic field from the machine’s magnetic motors large enough to overwhelm the inherent shielding of the IIs and cause distortion that is visible on the DSX video recording. To counteract this distortion effect, the magnetic field needs to be redirected away from the DSX testing space. As such, this study details the development and evaluation of a custom MuMETAL-lined box (Magnetic Shield Corporation, Bensenville IL, United States), fitted onto the eMTS to provideshielding and mitigate the effect of the magnetic field on the DSX imaging. MuMETAL is a nickel-iron ferromagnetic alloy with high permeability, used in applications to shield sensitive electronic equipment from low frequency magnetic fields by redirecting the field. The ferromagnetic shielding of the IIs in our DSX system is similar in composition to the proprietary MuMETAL purchased to cover the box and shield the IIs from the magnetic field produced by the eMTS (recommended field below 2 A/m). Once the magnetic field is redirected, the goal will be to perform cadaveric skin tissue testing using an eMTS inside a DSX testing space to quantify movement which should correlate to DSX image-derived tissue strain calculations during simulated gait (Maikos et al., 2024). The strain from mechanical testing is derived based on the circular sample test geometry, the location of the compressive load application, and the constraints applied by tissue fixture clamps.

## 2 Materials and equipment

### 2.1 Mechanical testing system cart design

A custom steel cart (MTS Systems Corporation, Eden Prairie, MN, United States) with dimensions of 29″ D X 29″ H X 14″ W was designed to support the load frame of the eMTS (MTS Acumen 3AT, MTS Systems Corporation, Eden Prairie, MN, United States) within the DSX volume. The cart was equipped with locking casters and risers a manual hydraulic lifting assembly with locking clamps, to raise and lower the cart height within 12 inches, permitting adjustment of the testing setup to accommodate the necessary angle to visualize the markers on the skin.

### 2.2 Shield box design

**FIGURE 2:**
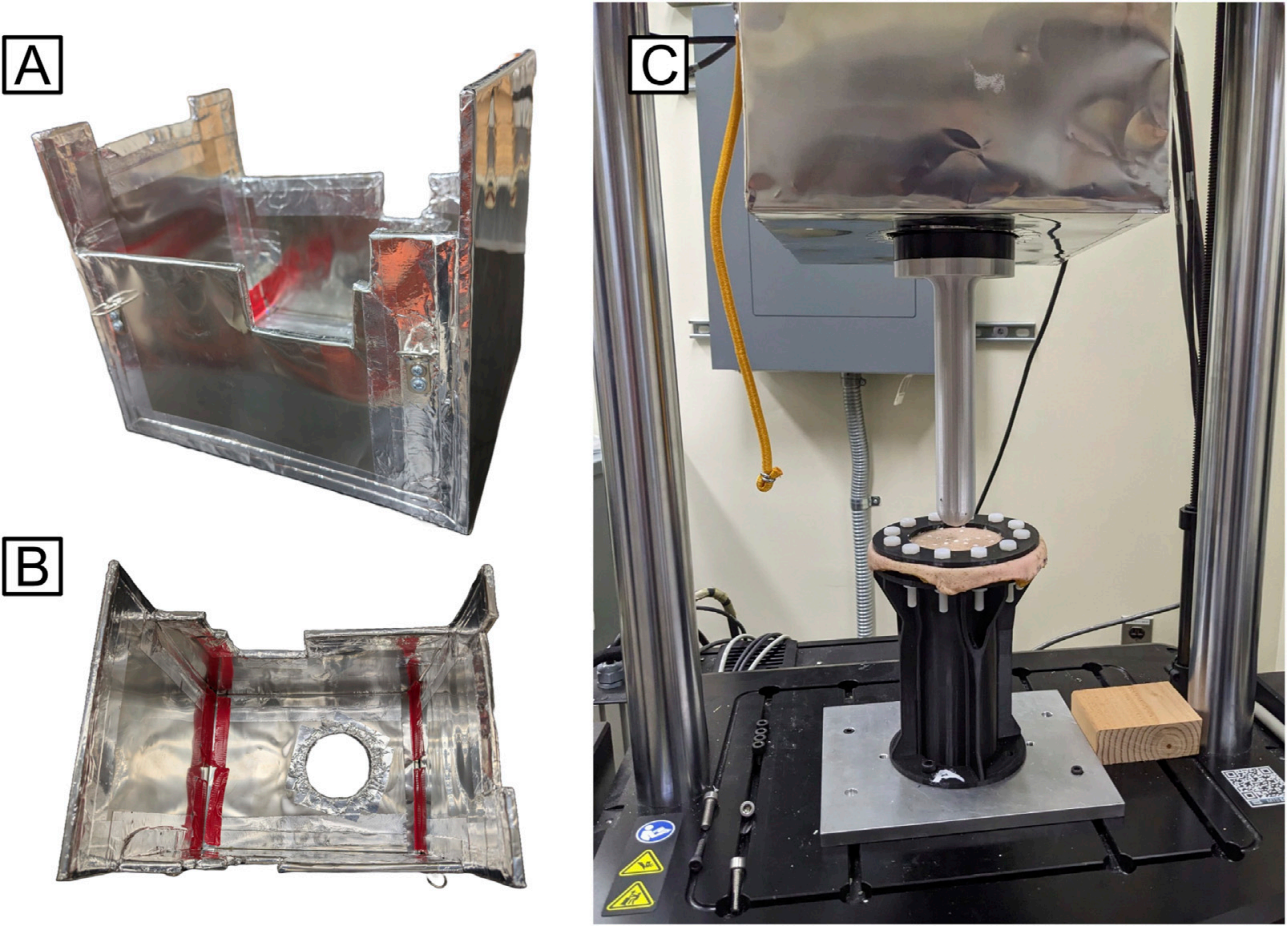
**(A)** Isometric and **(B)** top-down views of MuMETAL-lined shield box design; **(C)** Shield box interfaced with the mechanical testing system (to test cadaveric skin sample MTS Acumen 3AT).

A 3/8-inch thick plywood box was designed and fabricated to directly contour to the frame of the mechanical testing system and fully cover the magnetic motors. The plywood box was covered with 0.6 mm thick MuMETAL foil to reduce escape of the magnetic field (Figure 2). Magnetic flux recordings indicated that the highest magnetic field was at the bottom of the motor. Therefore, the bottom of MuMETAL box was tightly fitted to the eMTS load cell and magnetic motor. The eMTS motor housing was 23 cm in diameter and had an approximate length of 23 cm. The MuMETAL box had a width of 25 cm and a depth of 24 cm to accommodate the eMTS motor. The length of the MuMETAL box was 43 cm so as to be supported from the eMTS loadframe crossbar. A 10.5 cm diameter hole was made in the bottom of the MuMETAL box to allow the 10.3 cm diameter load cell with attached fixture plate to freely move in the test space. Prior to shielding, the highest magnetic reading recorded by a magnetic flux detector was 0.12 mT (1.2 Gauss). After fitting the MuMETAL-lined box to the eMTS, the magnetic reading was 0 mT.

## 3 Methods

### 3.1 Determining the accuracy and precision of the DSX system

Marker-based and 3D volumetric model-based tracking have been characterized to establish precision and accuracy for several DSX systems (Bey et al., 2008; Brainerd et al., 2010). However, the marker-based precision and accuracy of the DSX system used for this study have not been measured.

**FIGURE 3:**
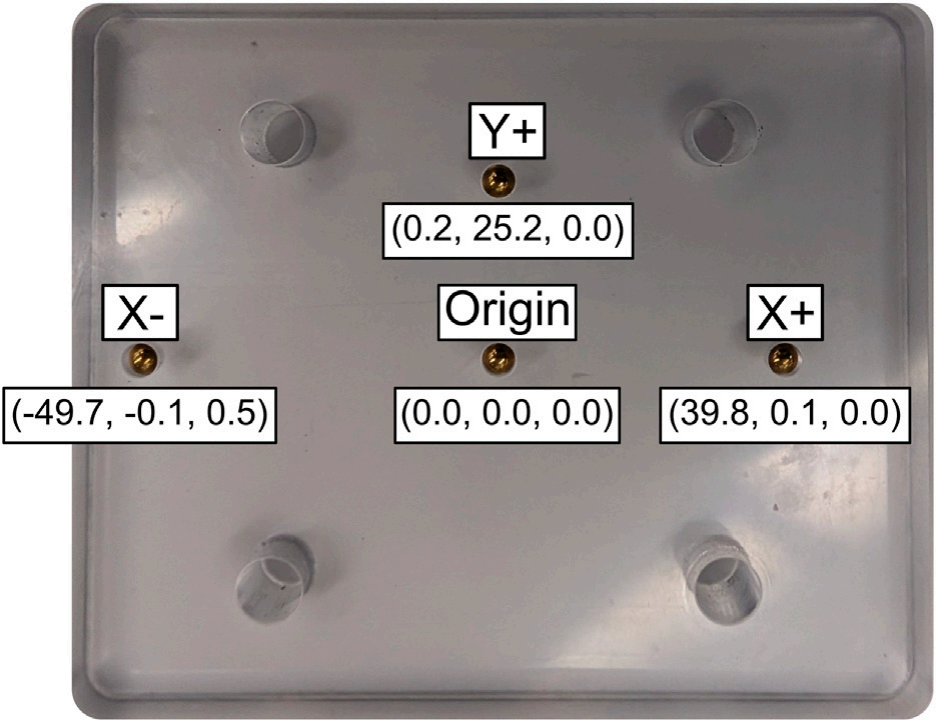
Precision-machined polycarbonate object with embedded beads. The CT-based reference bead locations are given in a local coordinate system (X, Y, Z) relative to the origin.

To determine the marker-based tracking accuracy associated with the DSX system, a polycarbonate object (PcO) was CNC-machined with 4.76 mm diameter brass beads (McMaster Carr, Inc.) embedded at known distances (Figure 3), with a standard machining tolerance of 0.13 mm. This object was CT scanned prior to testing in the DSX system to determine absolute bead positions. The absolute CT-based bead positions were located using the three planar views (x, y, z) to account for any displacement artifact that may have occurred during the press fitting of the brass beads. The centroid locations were used as reference landmarks to track the DSX imaged-derived bead positions during testing. The angle between the two IIs was set at 60°. The PcO was attached to a modified electromechanical stance simulator (Maikos et al., 2024) that was designed to interface with the DSX system for cadaveric tissue testing. The framework of the stance simulator consisted of two stands with a linear track spanning the capture volume of the DSX system. A stepper motor with a built-in ACME lead screw was modified to permit the PcO to be mounted to the flange nut of the ACME screw. This setup permitted remote linear translation and axial rotation of the PcO. The polycarbonate object was imaged in the DSX system during two tests: Linear translation (125 mm at 40 mm/s) and axial rotation (4.3 rad/s). The distance between beads during both linear translation and axial rotation was measured using DSX marker tracking in 1,000 sequential frames of video, recorded at 100 frames per second for each trial.

Using the origin, +Y, and +X bead locations, a local coordinate system (CS) was created on the plate, and the bead location data were transformed relative to this CS. The 3D data was not filtered prior to calculating accuracy and precision. Accuracy was calculated as the mean difference between the measured pairwise distances during DSX marker tracking from the CT-based reference bead distances. Precision was calculated as the standard deviation of the pairwise distances between the markers (Tashman and Anderst, 2003). Root mean square (RMS) error was also calculated to determine variance of the bead locations in the local CS relative to the CT-based reference bead positions (Equation 1). RMS error represents the square root of the average squared difference between the expected and observed outcome, ${\hat{y}}_{i}$ and $y_{i}$ respectively, where n is the total number of observations. Any positional variance from the reference data would therefore be considered DSX marker tracking error, also considered the intrinsic error of the DSX system.

$$RMS\;Error=\sqrt{\sum_{i=1}^{n}\frac{{\left({\hat{y}}_{i}-y_{i}\right)}^{2}}{n}}$$ (1)

### 3.2 Mitigation of magnetic field distortion

**FIGURE 4:**
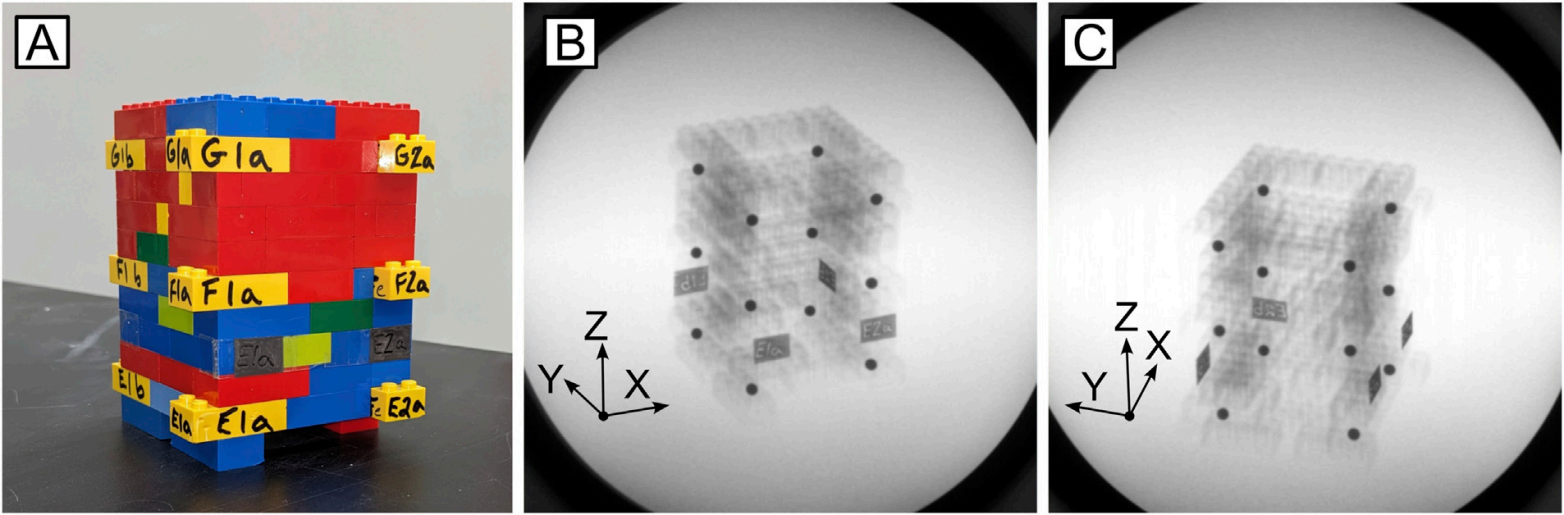
Calibration cube utilized to calculate RMS error associated with the mechanical testing system-induced magnetic field. **(A)** Image of the LEGO cube; **(B)** Inline and **(C)** offset x-rays of known bead locations.

To analyze the effects of the magnetic field interference on DSX tracking measurements, a rectangular, cuboid calibration object (Figure 4) constructed of LEGO bricks (LEGO Group, Billund, Denmark) was assembled with twelve, 5 mm diameter metallic beads press-fit into individual bricks, spaced at known, regular intervals. The precise manufacturing tolerance (±0.002 mm) of LEGO bricks makes the assembled cube a reconfigurable calibration device that is within the known accuracy tolerances of typical DSX systems (Miranda et al., 2011).

The calibration object was placed on the eMTS inside the DSX capture volume with the IIs angled at 60°. The custom MuMETAL lined box was placed on the mechanical testing system to shield the eMTS motor. For image distortion testing the MTS was positioned at either the minimum (65 cm) or maximum (103 cm) distance from the IIs with the calibration object located within the imaging space. DSX imaging was then collected with the eMTS powered on to create the magnetic field with the actuator moving axially at speeds of 0.1, 0.5, 1.0, 5.0, or 10.0 mm/s at each distance. The calibration object did not interact with the eMTS actuator. With no contact between the actuator and the calibration cube, any resultant image-derived bead movement that fell outside the calculated error associated with DSX marker tracking could therefore be attributed to image distortion caused by the eMTS magnetic field. Additionally, a controlled, static trial for each distance was collected in which the calibration object was placed on the unshielded, unpowered eMTS within the DSX capture volume. These conditions determined the effect of distance from the IIs and speed of the shielded actuator on the distortion of the DSX images. For each trial, 1,000 sequential frames of video were recorded at 100 frames per second.

The DSX marker position data were tracked and analyzed using DSX software (DSX, HAS-Motion, Ontario, Canada). For each trial, RMS error was calculated over 1,000 frames of the collected bead locations, relative to the position measured on the first frame of the recording. RMS error values represent the quantified accuracy of each recording and are used to determine the relationship of RMS error to the distance from the IIs and the speed of the actuator. These data were calculated for the positional magnitude of each bead, or the squared sum of its 3D coordinates, as defined by the local CS, maintaining consistency between trials collected between two test days. RMS error was then determined for each individual bead.

### 3.3 Statistics

The RMS error bead data were averaged for each group for each plane of motion. One Way Analysis of Variance was computed between groups with Tukey *post-hoc* comparisons between all groups using SPSS software (SPSS Inc., Chicago IL, United States). Statistical significance was set at 0.05.

## 4 Results

### 4.1 Accuracy and precision of the DSX system

**FIGURE 5:**
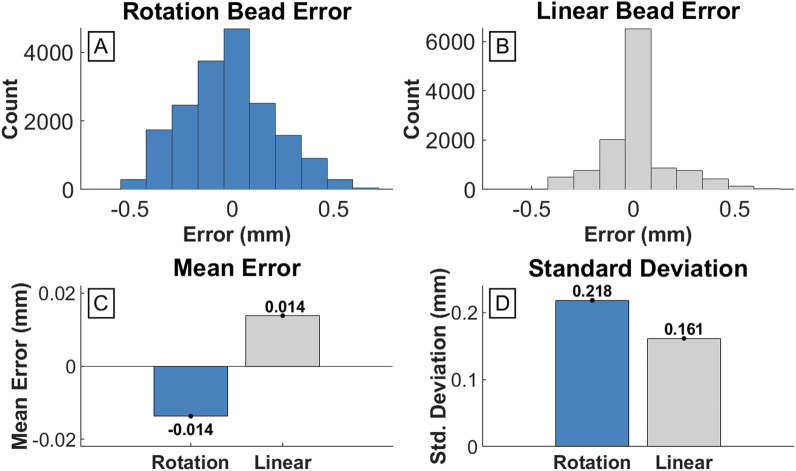
Error of inter-bead distances measured by DSX compared to known CT scan measurements. **(A)** and **(B)** are histograms detailing the frequency of error values for each inter-bead distance across all measured frames for the rotation and linear test, respectively. **(C)** Bar graph of the mean error for the rotation test (−0.014 mm) and linear test (0.014 mm). **(D)** Bar graph of the standard deviation of error for the rotation test (0.218 mm) and linear test (0.156 mm).

The PcO containing four press-fit brass markers was systematically moved within the DSX test space while capturing image recordings, to quantify the accuracy and precision of the DSX system. The known CT-based pairwise distances were compared to the 3D position of the markers measured by the DSX marker tracking system. The mean error for all pairwise inter-bead distances was 0.014 mm (±0.161 mm) during linear translation and −0.014 mm (±0.218 mm) during axial rotation (Figure 5). RMS error was also calculated to determine variance of the bead locations in the local CS relative to the reference CT-based bead positions (Table 1). Absolute inter-bead mean RMS errors were 0.12 (±0.12) mm during linear translation and 0.21 (±0.06) mm during axial rotation of the PcO.

**TABLE 1:** RMS errors for DSX marker tracking.

PcO Movement	RMS Error (mm)
Linear Translation	0.12 (0.12)
Axial Rotation	0.21 (0.06)

Values represent Mean and Standard Deviation in millimeters.

### 4.2 Error associated with distance from the IIs and speed of the actuator

The effects of distance from the IIs and mechanical testing system actuator speed on the distortion of the DSX marker-based tracking were evaluated within the DSX test space. The motor of the mechanical testing system was shielded using a custom-designed MuMETAL box and the distance of the object to the IIs was positioned at either 65 cm or 103 cm for data collection. The speed of the MTS actuator was then systematically increased from 0.1 mm/s to 10 mm/s for each distance. It should be noted that no dynamic tests were performed in which the MTS motors were unshielded due to the potential for permanent damage to the IIs.

**FIGURE 6:**
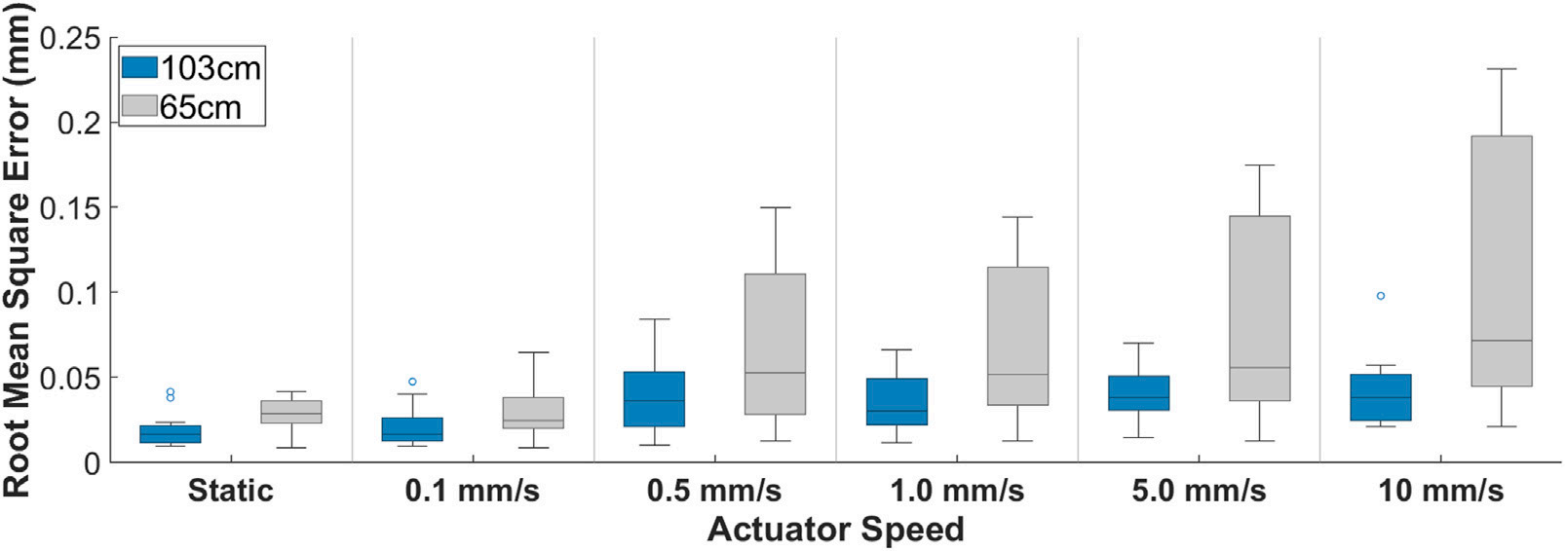
Root square mean error associated with changes in actuator speed of the mechanical testing machine at distances of 65 cm (gray) and 103 cm (blue) of the object to the image intensifiers.

At 65 cm from the IIs, for all actuator speeds less than 10 mm/s, the absolute mean RMS error of the positional magnitude was less than 0.10 mm and was not statistically different than the control test (static, unshielded) (p = 0.78) (Figure 6; Table 2). Absolute mean RMS error at an actuator speed of 10.0 mm/s (±0.110 mm) was statistically higher than the control test (p = 0.02; Table 2). At 103 cm from the IIs, the absolute mean RMS error for all markers was below 0.05 mm at all actuator speeds. Absolute mean RMS errors at actuator speeds ≥5.0 mm/s were significantly higher than the control condition (p < 0.05) (Table 2). All RMS errors at each actuator speed were below the known RMS error of the DSX system (0.12 mm in linear translation and 0.21 mm in axial rotation). In general, there was a proportional change in RMS error with increasing distance and decreasing speed (Figure 6; Table 2).

**TABLE 2:** Mechanical testing shielding RMS error.

65 cm object to IIs		
Actuator speed (mm/s)	RMS Error (mm), Mean (Std)	Sig
Control (Static, Unshielded)	0.028 (0.01)	
0.1	0.029 (0.02)	1.000
0.5	0.067 (0.05)	0.390
1.0	0.069 (0.05)	0.346
5.0	0.082 (0.06)	0.096
10.0	0.110 (0.08)	**0.002**

103 cm Object to IIs		
Actuator speed (mm/s)	RMSE, Mean (Std)	Sig
Control (Static, Unshielded)	0.019 (0.01)	
0.1	0.021 (0.01)	1.000
0.5	0.039 (0.02)	0.071
1.0	0.035 (0.02)	0.196
5.0	0.040 (0.02)	**0.041**
10.0	0.042 (0.02)	**0.017**

Bolded numbers indicate p-values less than 0.05.

## 5 Discussion

This study developed and tested a custom MuMETAL-lined box that was designed to interface with a mechanical testing system to eliminate the effect of image distortion when performing mechanical testing with a servo-electric mechanical testing system during DSX imaging. The primary challenge with the experimental setup was that the electromagnetic mechanical testing system created a magnetic field from the machine’s motors significant enough to overwhelm the inherent shielding of the IIs and caused unforeseen distortion of the DSX images. Distortion was observed primarily during motor acceleration and deceleration phases and was primarily visible during DSX imaging without the MuMETAL lining at both the onset and end of the test runs. Additionally, the speed of the actuator affected the distortion as higher speeds distorted the DSX images to a greater extent. Implementation of a servo hydraulic machine could have circumvented this issue, but this was not practical within the DSX setup. To measure the effect of the custom MuMETAL box on the redirecting of the magnetic field generated by the electrodynamic mechanical testing system, experiments were conducted to determine the magnitude of the distortion on the DSX images. Any error caused by the distortion of the magnetic field was then compared across testing conditions to ensure the error fell within an accepted margin of error associated with the DSX system.

### 5.1 DSX marker tracking error

The accuracy of the DSX marker tracking system was measured using a machined PcO with brass beads placed at known distances. The bead positions were then tracked using DSX imaging during translation and axial rotation of the object and pairwise distances were compared to the known bead distances. The mean measured inter-bead distances (0.014 mm during translation and −0.014 mm during rotation) were within the standard tolerance of the PcO (Figure 3A). Both the accuracy and precision were similar to other reported DSX systems (Brainerd et al., 2010; Miranda et al., 2011). Using a similar DSX system, Brainerd et al. reported a mean absolute error of 0.037 mm (Brainerd et al., 2010) while Miranda et al. measured systemic errors of 0.1–0.25 mm for marker-based tracking (Miranda et al., 2011). DSX marker-based tracking error primarily involves digitizing “error” of marker centroid positions on DSX images (Brainerd et al., 2010). Clear markers on DSX images can reduce this type of digitizing error, but even subtle deviations from the absolute centroid can cause slight tracking error. Additionally, during fabrication of the PcO, the tolerance in placement of the origin bead can compound any error during pairwise bead measurement. For this study, the error associated with DSX tracking was compared with the magnetic field mitigation technique to determine effectiveness of eliminating DSX image distortion.

### 5.2 Mitigation of DSX image distortion

This study evaluated the effect of shielding the magnetic field on DSX image distortion by comparing the mitigation technique with both a control condition, where no magnetic field was generated (i.e., the mechanical testing system was unpowered), and the error associated with the DSX tracking measurement. The design included a wooden box lined with 0.6 mm MuMETAL foil thickness. At the minimum distance to the IIs (65 cm), higher actuator speeds (10 mm/s) produced RMS errors up to 0.11 mm, which were significantly higher than the control test. When the distance was increased to the maximum distance to the IIs (103 cm), all RMS errors were below 0.05 mm for all speeds. The results of this study indicated a proportional change in RMS errors with increasing distance and decreasing speed. However, while closer distance to the IIs and higher actuator speeds produced larger RMS errors, results of this study indicated that the RMS errors for all experimental conditions fell within the error of DSX marker tracking. Therefore, the custom MuMETAL box effectively eliminated any DSX image distortion caused by the mechanical testing system regardless of the distance to the IIs and actuator speed by redirecting the magnetic field away from the DSX capture volume. As such, it is expected that performing mechanical testing at distances between 65 cm and 103 cm to the IIs at the speeds used in this study will also be effective in mitigating DSX image distortion to accurately track markers during DSX testing.

### 5.3 Future research

This study provides critical information on mitigating the effects of a magnetic field on DSX image distortion for future research that aims to perform electromagnetic mechanical testing inside a DSX capture volume. DSX has been traditionally used to evaluate joint kinematics, joint impingement, and implant kinematics (Hoshino and Tashman, 2012; Farrokhi et al., 2015; Setliff and Anderst, 2024). More recently, DSX has been used to develop soft tissue models that assess soft tissue contacts, stresses, and strains (Gale et al., 2020; Kim-Wang et al., 2023). DSX offers the advantage of measuring *in-vivo* tissue strains with high levels of accuracy and repeatability (Bey et al., 2008; Bey et al., 2006; Brainerd et al., 2010; Zhang et al., 2013; Maikos et al., 2021) during dynamic activities, which would otherwise be unable to be measured using the traditional DSX experimental kinematics techniques (Hoshino and Tashman, 2012; Farrokhi et al., 2015; Setliff and Anderst, 2024). However, measuring tissue strain using DSX requires validation against gold standard measurements, which has yet to be performed and is a critical step prior to implementation in future research and clinical applications. As such, the test setup in this study will ultimately be used to apply displacements to cadaveric skin tissue (mimicking tissue deformation inside a prosthetic socket) in a DSX testing space as a method of validation for imaging-derived tissue strain calculations using DSX marker tracking. Validation can be performed via controllable and reproducible tensile or extrusion tissue testing using an electromagnetic mechanical testing system while simultaneously imaging radiopaque markers placed on the skin sample using DSX. By redirecting the magnetic field with a custom MuMETAL-lined box, the RMS errors indicated that future experiments can accurately measure changes in position within the DSX system (without image distortion) to validate DSX as a method of measuring *in vivo* tissue strain.

There are some limitations associated with this study. The study did not quantify the unshielded condition after the distortion was identified nor its perceived effect on the image intensifiers. This test condition was avoided to protect the IIs from progressive damage associated with magnetic field exposure. Additionally, only the minimum and maximum distances to the IIs were examined (65 and 103 cm) when evaluating the RMS errors. The rationale for not evaluating intermediate distances was the absence of quantifiable distortion at the minimum distance from the IIs, regardless of motor velocity. Polycarbonate is a non-metallic, thermally stable, and non-absorbent material suitable for its accuracy test application. However, the opacity of the PcO did not permit laser scanning to determine the precise bead location tolerances compared to the design specifications.

## Data Availability

The raw data supporting the conclusions of this article will be made available by the authors, without undue reservation.

## References

[B1] Bey M. J. Kline S. K. Tashman S. Zauel R. (2008). Accuracy of biplane x-ray imaging combined with model-based tracking for measuring *in-vivo* patellofemoral joint motion. J. Orthop. Surg. Res. 3, 38. 10.1186/1749-799x-3-38 18771582 PMC2538511

[B2] Bey M. J. Zauel R. Brock S. K. Tashman S. (2006). Validation of a new model-based tracking technique for measuring three-dimensional, *in vivo* glenohumeral joint kinematics. J. Biomech. Eng. 128, 604–609. 10.1115/1.2206199 16813452 PMC3072582

[B3] Brainerd E. L. Baier D. B. Gatesy S. M. Hedrick T. L. Metzger K. A. Gilbert S. L. (2010). X-ray reconstruction of moving morphology (XROMM): precision, accuracy and applications in comparative biomechanics research. J. Exp. Zool. A Ecol. Genet. Physiol. 313, 262–279. 10.1002/jez.589 20095029

[B4] Dall’Ara E. Bodey A. J. Isaksson H. Tozzi G. (2022). A practical guide for *in situ* mechanical testing of musculoskeletal tissues using synchrotron tomography. J. Mech. Behav. Biomed. Mater 133, 105297. 10.1016/j.jmbbm.2022.105297 35691205

[B5] Farrokhi S. Meholic B. Chuang W. N. Gustafson J. A. Fitzgerald G. K. Tashman S. (2015). Altered frontal and transverse plane tibiofemoral kinematics and patellofemoral malalignments during downhill gait in patients with mixed knee osteoarthritis. J. Biomech. 48, 1707–1712. 10.1016/j.jbiomech.2015.05.015 26087880 PMC4521908

[B6] Gale T. Yang S. Mcgough R. Fiedler G. Anderst W. (2020). Motion of the residual femur within the socket during gait is associated with patient-reported problems in transfemoral amputees. J. Biomech. 112, 110050. 10.1016/j.jbiomech.2020.110050 33035840

[B7] Geerligs M. Van Breemen L. Peters G. Ackermans P. Baaijens F. Oomens C. (2011). *In vitro* indentation to determine the mechanical properties of epidermis. J. Biomech. 44, 1176–1181. 10.1016/j.jbiomech.2011.01.015 21296353

[B8] Graham H. K. Mcconnell J. C. Limbert G. Sherratt M. J. (2019). How stiff is skin? Exp. Dermatol 28 (Suppl. 1), 4–9. 10.1111/exd.13826 30698873

[B9] Griffin M. Premakumar Y. Seifalian A. Butler P. E. Szarko M. (2016). Biomechanical characterization of human soft tissues using indentation and tensile testing. J. Vis. Exp., 54872. 10.3791/54872 28060331 PMC5226394

[B10] Hoshino Y. Tashman S. (2012). Internal tibial rotation during *in vivo*, dynamic activity induces greater sliding of tibio-femoral joint contact on the medial compartment. Knee Surg. Sports Traumatol. Arthrosc. 20, 1268–1275. 10.1007/s00167-011-1731-6 22041716

[B11] Jor J. W. Parker M. D. Taberner A. J. Nash M. P. Nielsen P. M. (2013). Computational and experimental characterization of skin mechanics: identifying current challenges and future directions. Wiley Interdiscip. Rev. Syst. Biol. Med. 5, 539–556. 10.1002/wsbm.1228 23757148

[B12] Kim-Wang S. Y. Bradley P. X. Cutcliffe H. C. Collins A. T. Crook B. S. Paranjape C. S. (2023). Auto-segmentation of the tibia and femur from knee MR images via deep learning and its application to cartilage strain and recovery. J. Biomech. 149, 111473. 10.1016/j.jbiomech.2023.111473 36791514 PMC10281551

[B13] Maikos J. T. Chomack J. M. Herlihy D. V. Paglia D. N. Wetterstrand C. O'Connor J. P. (2024). Quantifying bone and skin movement in the residual limb-socket interface of individuals with transtibial limb loss using dynamic stereo X-ray: protocol for a lower limb loss cadaver and clinical study. JMIR Res. Protoc. 13, e57329. 10.2196/57329 38669065 PMC11087852

[B14] Maikos J. T. Chomack J. M. Loan J. P. Bradley K. M. D’Andrea S. E. (2021). Effects of prosthetic socket design on residual femur motion using dynamic stereo X-ray - a preliminary analysis. Front. Bioeng. Biotechnol. 9, 697651. 10.3389/fbioe.2021.697651 34447740 PMC8383143

[B15] Miranda D. L. Schwartz J. B. Loomis A. C. Brainerd E. L. Fleming B. C. Crisco J. J. (2011). Static and dynamic error of a biplanar videoradiography system using marker-based and markerless tracking techniques. J. Biomech. Eng. 133, 121002. 10.1115/1.4005471 22206419 PMC3267989

[B16] Ni Annaidh A. Bruyere K. Destrade M. Gilchrist M. D. Ottenio M. (2012). Characterization of the anisotropic mechanical properties of excised human skin. J. Mech. Behav. Biomed. Mater 5, 139–148. 10.1016/j.jmbbm.2011.08.016 22100088

[B17] Ottenio M. Tran D. Ni Annaidh A. Gilchrist M. D. Bruyere K. (2015). Strain rate and anisotropy effects on the tensile failure characteristics of human skin. J. Mech. Behav. Biomed. Mater 41, 241–250. 10.1016/j.jmbbm.2014.10.006 25455608

[B18] Papaioannou G. Mitrogiannis C. Nianios G. Fiedler G. (2010). Assessment of amputee socket-stump-residual bone kinematics during strenuous activities using Dynamic Roentgen Stereogrammetric Analysis. J. Biomech. 43, 871–878. 10.1016/j.jbiomech.2009.11.013 20047746

[B19] Remache D. Caliez M. Gratton M. Dos Santos S. (2018). The effects of cyclic tensile and stress-relaxation tests on porcine skin. J. Mech. Behav. Biomed. Mater 77, 242–249. 10.1016/j.jmbbm.2017.09.009 28954243

[B20] Salter D. C. Mcarthur H. C. Crosse J. E. Dickens A. D. (1993). Skin mechanics measured *in vivo* using torsion: a new and accurate model more sensitive to age, sex and moisturizing treatment. Int. J. Cosmet. Sci. 15, 200–218. 10.1111/j.1467-2494.1993.tb00075.x 19272125

[B21] Sandford E. Chen Y. Hunter I. Hillebrand G. Jones L. (2013). Capturing skin properties from dynamic mechanical analyses. Skin. Res. Technol. 19, e339–e348. 10.1111/j.1600-0846.2012.00649.x 22672357

[B22] Setliff J. C. Anderst W. J. (2024). A scoping review of human skeletal kinematics research using biplane radiography. J. Orthop. Res. 42, 915–922. 10.1002/jor.25806 38366965

[B23] Tashman S. Anderst W. (2003). *In-vivo* measurement of dynamic joint motion using high speed biplane radiography and CT: application to canine ACL deficiency. J. Biomech. Eng. 125, 238–245. 10.1115/1.1559896 12751286

[B24] Tran H. V. Charleux F. Rachik M. Ehrlacher A. Ho Ba Tho M. C. (2007). *In vivo* characterization of the mechanical properties of human skin derived from MRI and indentation techniques. Comput. Methods Biomech. Biomed. Engin 10, 401–407. 10.1080/10255840701550287 17891674

[B25] Zhang J. Lv L. Shi X. Wang Y. Guo F. Zhang Y. (2013). 3-D reconstruction of the spine from biplanar radiographs based on contour matching using the Hough transform. IEEE Trans. Biomed. Eng. 60, 1954–1964. 10.1109/tbme.2013.2246788 23412567

